# A Systematic Review on Microbial Profiling Techniques in Goat Milk: Implications for Probiotics and Shelf-Life

**DOI:** 10.3390/ijms26125551

**Published:** 2025-06-10

**Authors:** Nare Jessica Monareng, Keabetswe T. Ncube, Charles van Rooi, Mamokoma C. Modiba, Bohani Mtileni

**Affiliations:** 1 Department of Animal Sciences, Tshwane University of Technology, Pretoria 0001, South Africa; narejessicamonareng@gmail.com (N.J.M.); mtilenib@tut.ac.za (B.M.); 2 Department of Biotechnology and Food Technology, Tshwane University of Technology, Pretoria 0001, South Africa; vanrooicf@tut.ac.za

**Keywords:** dairy science, goat milk, metagenomics, microbial diversity, probiotics, next generation sequencing, spoilage

## Abstract

Due to its high digestibility, rich nutrient profile, and potential probiotic content, goat milk is an essential nutritional resource, particularly for individuals with cow milk allergies. This review summarises the current state of microbial diversity in goat milk, emphasising the implications for quality, safety, and probiotic potential. This systematic review adhered to PRISMA guidelines, conducting a comprehensive literature search across PubMed, ScienceDirect, and Google Scholar using keywords related to microbial profiling in goat milk. The inclusion criteria targeted English-language studies from 2000 to 2025 that utilised high-throughput or next-generation sequencing methods. Out of 126 articles screened, 84 met the eligibility criteria. The extracted data focused on microbial diversity, profiling techniques, and their respective strengths and limitations in evaluating probiotic potential and spoilage risks. The review addresses the challenges linked to microbial spoilage and the composition and functional roles of microbial communities in goat milk. With species such as *Bacillus* and *Pseudomonas* playing crucial roles in fermentation and spoilage, key findings emphasise the prevalence of microbial phyla, including *Proteobacteria*, *Firmicutes*, and *Actinobacteria* in goat milk. The review also explores the probiotic potential of the goat milk microbiota, highlighting the health benefits associated with strains such as *Lactobacillus* and *Bifidobacterium*. Significant discoveries underline the necessity for advanced multi-omics techniques to thoroughly define microbial ecosystems and the substantial gaps in breed-specific microbiota research. Important findings illustrate the need for enhanced multi-omics techniques, given the challenges of host RNA and protein interference, low microbial biomass, and limited goat-specific reference databases, for optimising probiotic development, spoilage prevention strategies, and integrating metagenomics, metabolomics, metaproteomics, and metatranscriptomics to improve milk quality and safety as some of the future research objectives. This study emphasises the importance of understanding goat milk microbiology to advance dairy science and enhance human health.

## 1. Introduction

Given its high digestibility, rich nutrient profile, and microbial content, goat milk has long been recognised as an essential nutritional resource, especially for those allergic to cow’s milk. Goat milk, a staple of dairy production in many areas, makes a substantial contribution to the global dairy market, rural livelihoods, and food security. Its production represents 13.5% of total non-bovine milk production, making it one of the most widely produced non-bovine milks worldwide [1]. The demand and production of goat milk are increasing due to its potential to replace bovine milk consumption. This is attributed to its non-allergenic nature, high digestibility, nutritional superiority, and medicinal properties [2]. Goat milk’s microbial makeup, however, has two functions: either it may improve the milk’s nutritional value and probiotic potential, or it can cause spoiling and food safety issues, which presents serious problems for both producers and consumers.

Numerous factors, such as breed, environmental conditions, farm management techniques, and lactation stage, affect the microbial diversity in goat milk [3,4]. *Proteobacteria*, *Firmicutes*, *Bacteroidetes*, and *Actinobacteria* are among the dominant microbial phyla that have been found; *Lactobacillus*, *Pseudomonas*, and *Bifidobacterium* are among the genera that are important for fermentation, spoiling, and probiotic potential [5,6]. It has been shown that [3,7], spoilage organisms like *Pseudomonas* and *Bacillus* can drastically shorten the shelf life of goat milk and damage its quality, whereas *Lactobacillus* and *Bifidobacterium*, two good microorganisms, add to their health-promoting qualities. Strains like *Lactobacillus plantarum* from goat milk exhibit acid/bile tolerance, a key probiotic trait enabling survival in the human gastrointestinal (GI) tract [8]. Furthermore, the discovery of pathogens such as *Chlamydia abortus* and *Listeria monocytogenes* in goat milk emphasises how crucial it is to comprehend and manage microbial contamination to guarantee food safety [9,10].

Beyond ensuring basic dairy safety, microbial profiling is crucial, especially in areas where traditional fermented dairy products are consumed in large quantities. Naturally fermented milk products derived from goat and cow milk are an essential part of the diets of people in Southern Africa [11]. While goat milk is known for its rich microbial diversity, including *Lactobacillus* spp., there is a lack of direct comparative data between goat and cow milk. This gap highlights the need for further research. However, these investigations also discovered microorganisms linked to spoilage, such as *Bacillus* and *Pseudomonas*, highlighting the necessity of better microbial profiling methods to improve food safety. Knowledge on the microbial communities in conventional dairy products has improved recently, thanks to high-throughput next-generation sequencing; however, difficulties in attaining strain-level resolution and functional characterisation still exist, requiring more study into metagenomic and metabolomic applications in goat milk research.

The capacity to describe the intricate microbial communities in goat milk has been completely transformed by recent developments in microbial profiling methods, including 16S rRNA, shotgun metagenomics, metabolomics, metaproteomics, and metatranscriptomics. Researchers can now detect microbial communities at a previously unheard-of level of precision, thanks to high-throughput next-generation sequencing technologies, which also help them find biomarkers for probiotic production, spoilage prevention, and quality control [3,12]. For example, metabolomic analysis has discovered important compounds linked to milk quality and shelf-life, whereas metagenomic techniques have demonstrated the functional involvement of microbial communities in fermentation and spoilage [13,14]. Even with these developments, there are still a lot of unanswered questions about the functional relationships between microbial communities, breed-specific microbiota, and the use of multi-omics techniques to maximise goat milk production [15]. 

These knowledge gaps persist partly because each microbial profiling technique has inherent strengths and limitations that shape its utility for addressing specific research questions. Although culture-based techniques are vital and economical for separating viable probiotic strains, such as *Lactobacillus* [3]. They are unable to identify unculturable species that might affect the quality of milk. By sequencing all microbial DNA, metagenomics provides a comprehensive insight into microbial diversity; however, it is hindered by limitations in existing reference databases, leading to 60–80% of sequences remaining unclassified [12]. The necessity for better reference genomes that are adapted to dairy microbiomes is highlighted by this constraint.

Examining their applications demonstrates how complementary these methods are: metatranscriptomics shows how pathogens like *Pseudomonas* activate spoilage genes during refrigeration [16], while metabolomics detects spoilage biomarkers like biogenic amines with high sensitivity [13]. The specific tools and bioinformatics knowledge needed for NGS and multi-omics techniques, however, continue to be obstacles to their broad use [17].

The capacity to resolve breed-specific microbial interactions or enhance fermentation processes is a major issue that requires standardised, integrated methods in future research and is strongly impacted by these methodological limitations.

This systematic review aims to critically evaluate emerging microbial profiling techniques in goat milk research, focusing on their efficacy, applications, and limitations in detecting microbial diversity, spoilage organisms, and probiotic strains. Specifically, the review examines how tools such as 16S rRNA, shotgun metagenomics, metatranscriptomics, metabolomics, and metaproteomics contribute to understanding microbial functionality, improving milk safety, and enhancing probiotic potential. The key research questions guiding this review are as follows: What are the dominant microbial profiling techniques used in goat milk studies? How effective are these techniques in identifying probiotic and spoilage-associated microbes? What are the strengths and limitations of each technique in the context of goat milk microbiology? By addressing these questions, this review aims to inform future research, enhance microbial monitoring in dairy systems, and support the development of safer, functional goat milk products.

## 2. Results and Discussion

To gain a better understanding of the changing methodological landscape in goat milk microbiome research, Table 1 lists the most important microbial profiling methodologies found in the articles that were examined. Each method provides a comparative view of its significance in characterising microbial diversity, functional attributes, and potential probiotic or spoilage-associated bacteria in goat milk. Additionally, it details the underlying principles, advantages, and limitations of each method. This methodological overview sets the stage for interpreting the subsequent results.

### 2.1. Microbial Communities in Goat Milk

Goat milk harbours a diverse microbial community that significantly affects its quality, safety, and health benefits. These microbial communities vary depending on factors such as lactation stage, goat breed, farm management techniques and environmental conditions [3].

**Figure 1:**
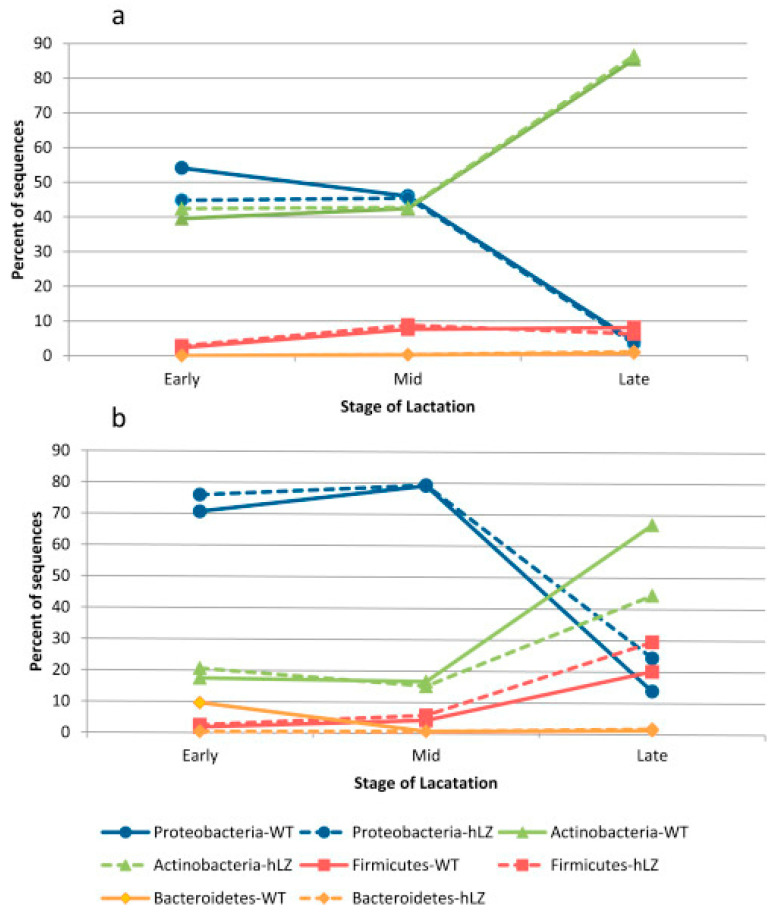
Dominant genera in wild-type and transgenic goat milk across early, mid, and late lactation stages. Reproduced from [4], using next-generation sequencing (NGS), amplicon sequencing (**a**) and clone-based sequencing (**b**). Reproduced with permission from [4], Food Microbiology, 46: 124. © Elsevier.

Among these factors, the lactation stage plays a critical role in shaping the microbial composition of goat milk. Studies have shown that microbial diversity shifts significantly from colostrum to late lactation, influencing milk safety and probiotic potential [4,18]. In colostrum, bacterial communities are dominated by genera such as *Lactobacillus*, which is commonly associated with probiotic potential [18]. As lactation progresses, there is an increase in microbial diversity, with *Proteobacteria* dominating at early and mid-lactation, and *Actinobacteria* becoming dominant at late lactation, which may influence milk quality and spoilage [4]. The shift in microbial composition across the early, mid, and late lactation stages is further shown in Figure 1 [4]. These changes highlight the dynamic nature of goat milk microbiota throughout lactation and suggest potential implications for dairy processing and probiotic applications.

The complexity of the goat milk microbiota, which is impacted by several variables and includes a dynamic balance of varied microbial communities, is highlighted by these changes in microbial composition throughout lactation. A systematic evaluation of the literature consistently identifies that goat milk is composed of a range of microbial phyla in goat milk microbiomes, with *Proteobacteria* [4], *Firmicutes* [18], *Bacteroidetes* [19], and *Actinobacteria* [5,6] being the most prevalent. A critical analysis of geographical patterns shows that *Proteobacteria* predominate (71.3%) in Chinese flocks [3], contrasting sharply with *Actinobacteria* dominance (62.8%) in Slovakian goat samples [5] and *Firmicutes* prevalence (67.5%) in Indian flocks [7]. These disparities reflect environmental and methodological influences: temperate climates seem to favour *Actinobacteria* development, tropical conditions correlate with increased *Proteobacteria*, and pasture-based systems show greater *Firmicutes* abundance compared to intensive operations. While methodological differences in sampling and analysis prevent definitive quantitative comparisons, the collective evidence suggests that regional production conditions significantly shape microbial profiles. This has important implications for dairy management, indicating that probiotic potential may be naturally enhanced in temperate systems, while tropical operations may require more rigorous spoilage mitigation strategies.

Significant geographical differences in the main microbial phyla are shown by a systematic comparison of investigations. For example, Slovakian goat milk samples had a greater abundance of *Actinobacteria* (62.8%) than Chinese goat milk samples, which were mostly constituted of *Proteobacteria* (71.3%) [3,5]. On the other hand, *Firmicutes* predominated in Indian samples (67.5%) [7]. These differences might be explained by sample or sequencing procedures, production techniques (pasture-based vs. intensive), and environmental factors (temperature, for example). These trends imply that temperate climates favour *Actinobacteria*, while tropical systems show elevated *Proteobacteria* numbers, maybe because of greater ambient temperatures and hygiene issues. However, direct meta-analysis was not feasible due to methodological variability. Future research employing standardised protocols across diverse regions would help clarify these geographical patterns and their underlying drivers.

Table 2 summarises the key microbial genera found in goat milk, their functional roles, and their impact on milk quality and probiotic potential. The relative abundance of these genera often differs across studies. For instance, key genera in a study conducted by [6] reported *Lactobacillus*, *Leuconostoc*, and *Streptococcus,* while another study found that the dominant genera included *Curtobacterium*, *Staphylococcus aureus*, *Bifidobacterium*, and *Streptococcus* [5]. A study by [3] found *Enterobacter* as the most abundant genus, indicating a high presence of environmental microbes. This study also identified the probiotic bacteria *Lactococcus* and pathogenic bacteria *Staphylococcus,* which suggests that goat milk microbiota includes both beneficial and harmful species, emphasising the need for proper handling and storage to maximise beneficial bacteria and minimise contamination [3].

**Figure 2:**
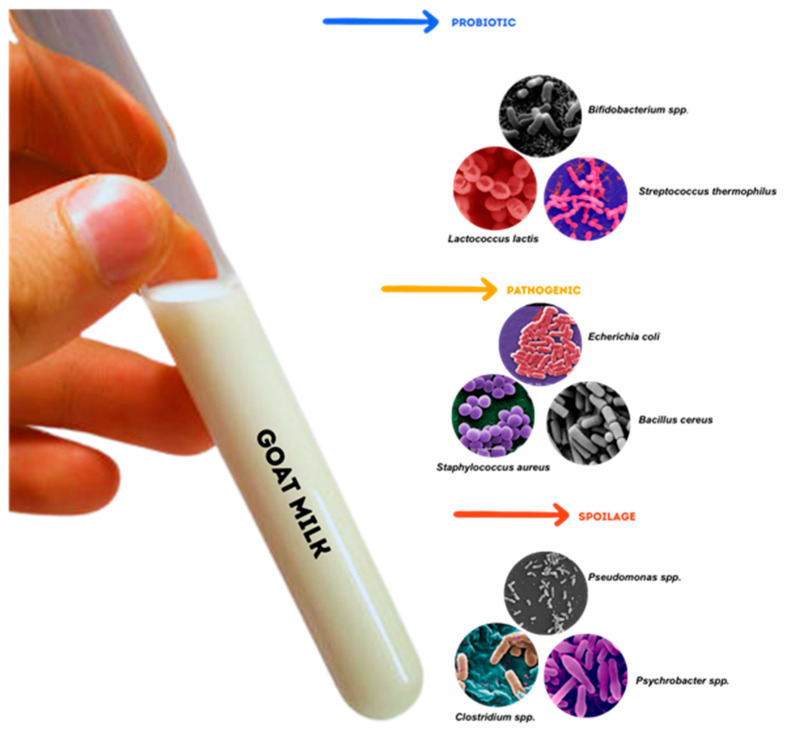
Goat milk contains various microbial groups that can be classified into three main categories: probiotics, spoilage organisms, and pathogens. Probiotic species, such as *Lactococcus lactis, Streptococcus thermophilus*, and *Bifidobacterium* spp., play crucial roles in fermentation processes and in promoting gut health. Pathogenic bacteria, such as *Staphylococcus aureus, Bacillus cereus*, and *Escherichia coli,* pose significant food safety risks. Spoilage organisms, including *Pseudomonas* spp. *Clostridium* spp., and *Psychrobacter* spp. are associated with the deterioration of milk quality. Image sources: Wikimedia Commons (n = 6); Science Photo Library (n = 2); JGI Genome Portal (n = 1) Figure compiled using Canva.

A greater number of potentially pathogenic bacteria were detected in goat milk samples, with *Pseudomonas* being the most abundant. Figure 2 provides a visual summary of key bacterial genera commonly identified in goat milk, grouped into three functional categories: probiotics, spoilage organisms, and pathogens. These microbes differ not only in morphology but also in their impact on product quality, safety, and functionality. For example, *Lactococcus lactis* and *Bifidobacterium* spp. contribute positively through fermentation and probiotic effects, while *Pseudomonas* spp. and *Bacillus* spp. are associated with spoilage, particularly under cold storage conditions. Pathogens such as *Staphylococcus aureus* and *Escherichia coli* are of concern due to their potential to cause foodborne illness and mastitis in dairy animals.

The makeup of the milk microbiota is significantly influenced by the health of the goat. A significant rise in opportunistic infections, including *Staphylococcus aureus, Escherichia coli, Mycoplasma* spp., and *Enterococcus* spp., is consistently linked to both subclinical and clinical mastitis [20]. Usually, these diseases increase somatic cell numbers, decrease microbial diversity, and upset the delicate balance between good and bad bacteria. Furthermore, although additional study is required, recent findings indicate that immunological characteristics particular to a breed may affect microbial resistance and susceptibility to dysbiosis. Future research must contextualise microbial alterations and their effects on dairy quality and safety by using goat health markers, such as clinical state or somatic cell count.

Additionally, the microbial profile of goat milk can also be influenced by the health status of the animal. For instance, studies have shown that milk from goats with mastitis often contains *Mycoplasma* sp., *Staphylococcus* spp., and *Escherichia* sp. *Shigella* sp. and *Enterococcus* sp. [20]. This shows a distinct microbial profile compared to healthy animals, with an increased prevalence of pathogenic bacteria. These findings highlight how health conditions, such as mastitis, can alter milk’s microbial diversity and impact its quality and safety. Furthermore, these results demonstrate the diversity of the microbial communities in goat milk, which affect not only the milk’s quality but also its potential for probiotics and spoilage.

The microbial ecosystem of goat milk extends beyond bacteria to include yeasts like *Kluyveromyces lactis* and fungi such as *Candida* species, which play roles in fermentation and spoilage [21,22]. However, this review focuses primarily on bacterial communities due to their dominant influence on milk quality, shelf-life and safety. The consistent identification of *Proteobacteria*, *Firmicutes*, and *Actinobacteria* across studies carries significant practical implications for dairy production. For instance, the high prevalence of *Lactobacillus* within *Firmicutes* in pasture-based systems suggests these herds may be ideal sources for probiotic starter cultures, particularly given this genus’ demonstrated acid and bile tolerance [8].

Conversely, the frequent detection of *Pseudomonas* in intensive farming operations highlights the need for enhanced hygiene protocols and temperature control during processing and storage. Regional variations in microbial profiles further suggest that breed selection and management practices could be optimised to enhance milk’s natural preservation qualities or probiotic potential. These insights enable dairy producers to tailor their approaches based on herd characteristics and production goals, aiming to develop functional fermented products or extend shelf-life.

Table 2 shows an overview of the principal bacterial groups identified in goat milk and delineates their respective roles in shaping milk quality, safety, and potential health benefits. The microbial ecosystem of goat milk represents a complex interplay among beneficial, spoilage-associated, and pathogenic bacteria, each exerting distinct functional influences on the dairy matrix. Beneficial bacteria, including *Lactococcus*, *Lactobacillus*, and *Leuconostoc*, are integral to milk fermentation processes, primarily through lactic acid production and the biosynthesis of bioactive metabolites. These genera contribute significantly to the development of organoleptic properties, enhancement of probiotic potential, and extension of product shelf-life.

Additionally, *Bifidobacteria* and *Curtobacteria* are linked to the production of short- and medium-chain fatty acids in fermented milk, which promote gastrointestinal health and support immune function in humans. On the other hand, spoilage bacteria such as *Pseudomonas*, *Bacillus*, and *Staphylococcus* produce extracellular enzymes that degrade milk proteins and lipids, compromising sensory qualities and reducing storage stability.

*Enterobacter* and some *Pseudomonas* species are also common in raw milk; their roles in both spoilage and potential fermentation need further investigation. Pathogenic bacteria, notably *Escherichia* spp. and *Staphylococcus aureus*, are particularly concerning due to their association with foodborne illnesses. Although some strains may be part of the commensal microbiota, their opportunistic pathogenicity poses significant public health risks.

**Figure 3:**
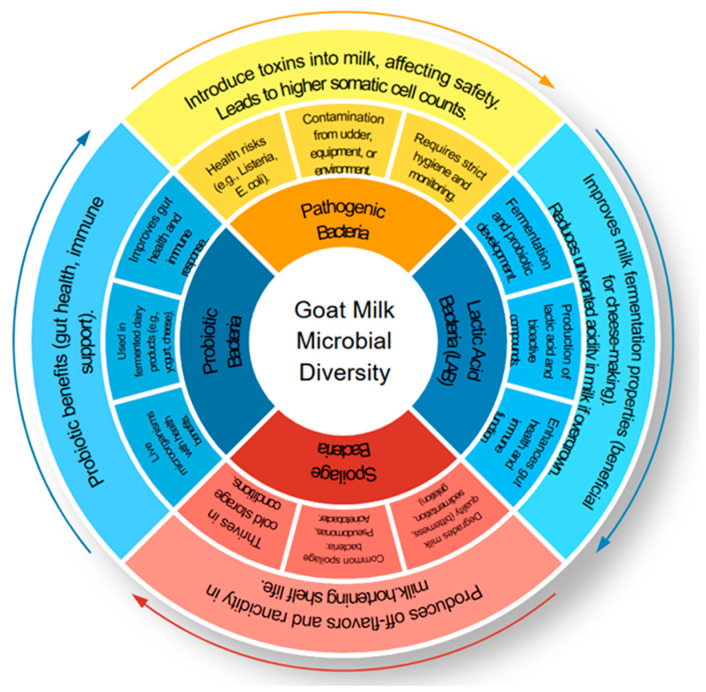
Schematic representation of microbial interactions in goat milk, illustrating the balance between beneficial microbes *(Lactobacillus*, *Bifidobacterium*, *Lactococcus*) that aid in fermentation and probiotic development, and harmful microbes (*Pseudomonas*, *Bacillus*, *Listeria monocytogenes*) associated with spoilage and food safety risks. Created using SlideModel’s Circular Process Diagram template (https://slidemodel.com), accessed 23 April 2025.

Furthermore, *Mycoplasma* spp., often linked to mastitis, reduces milk yield and alters microbial community composition, negatively affecting both safety and nutritional integrity. Figure 3 illustrates the microbial interactions in goat milk, highlighting the dynamic balance between beneficial and pathogenic microorganisms. The detection of *Staphylococcus aureus* serves as an indicator of poor hygienic conditions and broader microbial safety concerns [23]. Additionally, the presence of *Escherichia coli*, coliform bacteria, and *Clostridium tyrobutyricum* spores in the bulk tank and raw goat milk underscores the risk of contamination. Notably, studies have shown that cleaning teats with moist paper towels can significantly reduce microbial load [24]. On farms where hand milking was practised instead of automated systems, *S. aureus* was consistently identified, suggesting a strong correlation between farm management practices and pathogen prevalence [5].

Spoilage-associated bacteria such as *Pseudomonas* spp. produce extracellular enzymes that degrade milk proteins and fats, giving them a competitive advantage in raw milk environments. Their secretion of proteolytic enzymes is a key contributor to spoilage, particularly under cold storage conditions [2]. *Acinetobacter* spp., another group of spoilage organisms, are also capable of milk degradation and are known for their resilience across diverse environments [2].

In contrast, goat milk harbours beneficial lactic acid bacteria (LAB), with dominant strains including *Lactobacillus*, *Lactococcus*, and *Enterococcus* spp. [25]. Among these, *Lactococcus lactis* plays a pivotal role in milk fermentation. Certain LAB strains, such as *Lactobacillus paracasei*, exhibit strong probiotic potential by inhibiting pathogens like *E. coli* and *Listeria monocytogenes* through mechanisms such as biofilm suppression [25]. Their ability to survive acidic conditions further supports their application in fermented foods. Probiotic species, including *Streptococcus thermophilus*, *Lactococcus lactis*, and *Enterococcus* spp., have been shown to enhance host metabolism, support immune function, and regulate gut microbiota [26]. Additionally, LAB-derived bacteriocins demonstrate antimicrobial activity against both spoilage and pathogenic bacteria, positioning goat milk as a valuable source for bacteriocin discovery and probiotic development [26].

Therefore, optimising the microbial composition of goat milk by promoting beneficial taxa and mitigating the impact of spoilage and pathogenic organisms is crucial for improving milk quality, functional value, and safety. This underscores the need for advanced microbial monitoring and control frameworks, especially within raw milk value chains, to ensure both animal health and consumer protection.

Beneficial microbes such as *Lactobacillus*, *Bifidobacterium*, and *Lactococcus* contribute to the fermentation process, enhancing the probiotic potential and shelf-life of goat milk products by producing lactic acid and inhibiting pathogens. Conversely, spoilage organisms like *Pseudomonas* and *Bacillus* secrete heat-stable proteases and lipases that degrade milk proteins and fats, leading to bitterness, rancidity, and reduced shelf-life. Pathogenic microbes, including *Listeria monocytogenes*, *Staphylococcus aureus*, and *Chlamydia abortus*, pose serious food safety risks, underscoring the need for effective microbial monitoring.

### 2.2. Microbial Profiling Techniques

Accurately profiling microbial communities in goat milk is crucial for assessing its quality, safety, probiotic potential, and susceptibility to spoilage. Traditional culture-dependent methods have significant limitations on capturing the full diversity of microbial ecosystems, especially non-culturable and low-abundance species. This has led to a shift towards high-throughput, culture-independent approaches that offer a more comprehensive and precise understanding of microbial diversity.

Next-generation sequencing (NGS) technologies have been instrumental in this shift. Methods such as 16S rRNA gene sequencing and shotgun metagenomics allow researchers to identify microbial communities directly from milk samples without the need for culturing. High-throughput next-generation technologies enable the detection of both dominant and low-abundance taxa, provide strain-level resolution, and reveal functional gene content, which is particularly useful for identifying spoilage organisms and probiotic candidates. However, these techniques require significant bioinformatics resources and often struggle to distinguish between live and dead bacteria. According to a recent study [27], this limitation was highlighted, and integrating NGS with complementary techniques like metatranscriptomics and metabolomics was proposed to improve accuracy. Findings reported in [28] support this approach, suggesting that multi-omics integration enhances microbial resolution, while [29] demonstrate how different omics layers reveal microbial functions beyond genetic potential. Similar observations made in [30] also confirm that combining techniques strengthens microbial functional profiling. A comparison of the primary microbial profiling methods included in this study is shown in Table 3, which also highlights variations in front-end sample needs, resolution level, cost, and microbial detection sensitivity.

**Figure 4:**
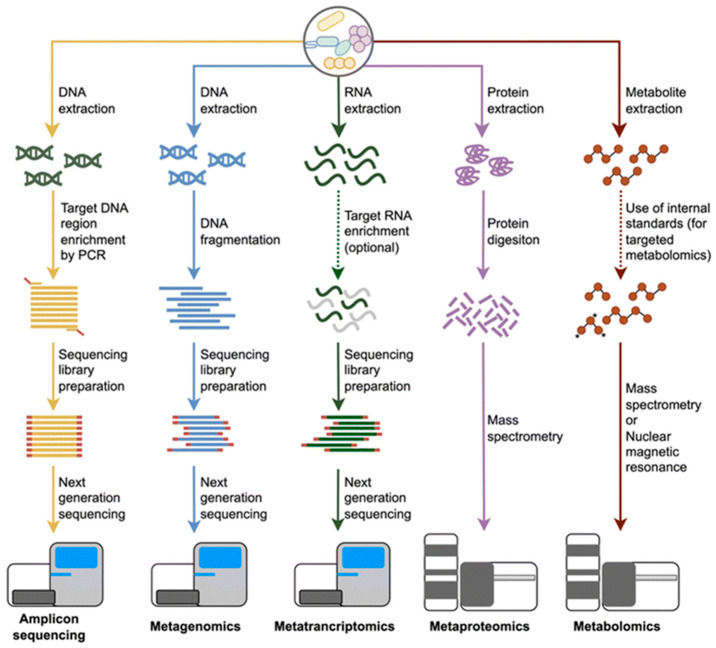
An overview of multi-omics approaches used in microbial community profiling includes workflows for amplicon sequencing, shotgun metagenomics, metatranscriptomics, metaproteomics, and metabolomics. The process begins with the extraction of DNA, RNA, proteins, or metabolites. Each omics method follows a distinct processing pipeline, from molecular extraction to sequencing or analytical techniques such as next-generation sequencing, mass spectrometry, or nuclear magnetic resonance (NMR). These methods collectively enable a comprehensive characterisation of microbial communities and their functional potential [28].

As the demand for high-quality, probiotic-rich dairy products continues to grow, understanding the microbial composition of goat milk is becoming increasingly important. Profiling techniques that go beyond simple taxonomic identification and explore microbial functions offer promising applications in improving fermentation processes, extending shelf life, and ensuring food safety. The following subsections will explore the key microbial profiling techniques used in goat milk research, highlighting their applications, advantages, and limitations (Figure 4).

#### 2.2.1. 16S rRNA Sequencing

The 16S rRNA sequencing is a popular amplicon-based method that targets conserved and variable regions within the bacterial 16S rRNA gene to enable taxonomic identification. It is widely used for profiling bacterial communities in complex ecosystems, including dairy products. This technique is particularly valuable in goat milk studies for identifying both dominant and rare bacterial taxa under various production or processing conditions. In a recent study [27], 16S rRNA sequencing benefits from standardised workflows and extensive reference databases, making it accessible and cost-effective. However, it has been shown that [30] it lacks resolution at the species or strain level, limiting its precision. Additionally, findings reported in [31] note that this method does not detect non-bacterial organisms such as yeasts or viruses.

**Table 3:** A simple comparative summary of microbial profiling techniques in goat milk analysis.

Technique	Cost Level	Level of Resolution	Microbial Detection Sensitivity	Sample Needs	Application
16S rRNA Sequencing	Low	Taxonomic identification at genus level; limited species resolution	Moderate	Moderate DNA yield	Used to study shifts in microbial diversity across lactation stages [4]; identification of *Lactobacillus plantarum* with probiotic traits [8]
Shotgun Metagenomics	High	High-resolution taxonomic profiling down to strain level; functional gene detection	High	High-quality, high-yield DNA	Detected antimicrobial resistance genes in *Staphylococcus* spp. in Brazilian goat milk [32]
Metatranscriptomics	Very high	Gene expression profiling of active microbial functions in real time	High	RNA integrity is crucial	Identified spoilage gene activation under cold storage [16]
Metabolomics	Medium-high	Functional protein identification: links taxa to metabolic pathways	Moderate-high	Metabolites-preserved samples	Tracked microbial protein markers in goat cheese spoilage and fermentation [33,34]
Metaproteomics	High	Detection of microbial metabolites reflects community functionality	Variable	Fresh, high-protein-yield samples	Assessed bioactive compounds (e.g., GABA, SCFAs) in goat milk kefir after fermentation and heating [35]

In goat milk studies, 16S rRNA sequencing has been utilised to investigate microbial shifts across different lactation stages [4] and to identify probiotic candidates and spoilage organisms such as *Lactobacillus* and *Pseudomonas* [3]. This technique has consistently produced complementary results, identifying dominant phyla like *Proteobacteria*, *Firmicutes*, and *Actinobacteria*. Several studies have reported shifts in microbial diversity throughout lactation [4,18], whereas others have noted variations in genus composition based on geographic location [3]. In a study conducted in South Africa [8], the *Lactobacillus plantarum* strain was identified using Amplicon sequencing. This strain was found in goat milk with probable probiotic characteristics like acid and bile tolerance as well as antimicrobial properties. Despite differences in sample sources and conditions, 16S rRNA sequencing reliably captures taxonomic trends.

Commonly used bioinformatic tools include QIIME and DADA2, which cluster sequences into operational taxonomic units (OTUs) or amplicon sequence variants (ASVs) [36,37]. While these tools are effective, their accuracy depends on the completeness and quality of reference databases. The limited availability of goat milk-specific reference data can affect taxonomic resolution.

#### 2.2.2. Shotgun Metagenomics

Shotgun metagenomics allows for the sequencing of all genomic material in a sample, offering both taxonomic and functional insights. This method captures strain-level variation and unculturable microbes, and it enables the detection of functional genes related to fermentation and spoilage. Shotgun metagenomics has been recognised for its capacity to uncover microbial interactions and functional regulation [30]. This is particularly relevant for goat milk, where microbial diversity can vary by breed, region, and processing conditions. While shotgun metagenomics offers numerous advantages, it is resource-intensive and cannot distinguish between viable and non-viable organisms [27]. The high costs and substantial bioinformatics requirements are also significant challenges [28]. However, integrating shotgun metagenomics with 16S rRNA sequencing has been proposed to improve microbial detection in complex samples [32]. Additionally, the limited availability of goat-specific functional databases remains a notable limitation [30].

The effectiveness of shotgun metagenomics in revealing microbial diversity and functionality in dairy environments has been demonstrated [12,17]. Although these studies primarily focused on various dairy matrices, their findings apply to goat milk due to microbial similarities. This technique consistently demonstrates its ability to detect unculturable microbes and novel gene functions. In Brazil, a study was conducted using shotgun metagenomics [38], PCR, as well as MALDI-TOF. Using these techniques, the multiresistant *Staphylococcus* spp. was identified, including strains harbouring the blaZ resistance gene, confirming the presence of virulence traits. However, shotgun metagenomics faces challenges in functional annotation due to incomplete databases, especially for goat-specific microbiota. Additionally, many studies have small sample sizes or concentrate on cow milk, leaving goat milk-specific functional pathways underexplored. There is also limited data on its application across diverse goat breeds and production systems.

Commonly used bioinformatic tools for taxonomic and functional classification include MetaPhlAn [39], MEGAHIT [40], and MG-RAST [41]. While these tools are effective for environmental and food microbiomes, accurate annotation requires careful database selection.

#### 2.2.3. Metatranscriptomics

Metatranscriptomics enables the functional profiling of microbial communities by sequencing the total RNA pool, capturing actively expressed genes at a given time. This technique allows researchers to assess microbial metabolism, stress responses, and community dynamics in response to external stimuli such as processing, disease, or environmental stressors. Metatranscriptomics is particularly useful for distinguishing active microbial functions from potential ones encoded in DNA, offering a dynamic and real-time view of the microbiome [27].

While metatranscriptomics provides powerful insights into microbial function, it faces limitations such as RNA instability, contamination from host transcripts, and complex sample preparation [42,43]. There are challenges in separating microbial RNA from the host tissue in animal-derived samples, which is a particularly relevant concern in goat milk studies [44,45,46]. Studies have demonstrated that metatranscriptomics can reveal dynamic microbial responses under various environmental and processing conditions.

For instance, spoilage genes activated during cold storage have been identified using a metatranscriptomics approach [16], while comparisons between metagenomic and transcriptomic outputs to better understand microbial activity during fermentation [47]. However, there is limited research using metatranscriptomics directly in goat milk. Further studies are needed to understand how gene expression varies across lactation stages, breeds, and processing conditions. Differentiating microbial RNA from host RNA remains a significant technical challenge.

Tools such as Salmon [44], Trinity [43], and DESeq2 [42] are commonly used for RNA quantification and differential expression analysis. These tools are powerful but require clean, high-quality RNA and careful filtering to separate host and microbial transcripts.

#### 2.2.4. Metaproteomics

Metaproteomics characterises the complete set of proteins expressed by microbial communities, providing direct evidence of metabolic activity and functional capability. This technique uniquely links microbial identity with function, identifying enzymes involved in fermentation, stress adaptation, and spoilage pathways. Metaproteomics captures what microbes are actively engaged in, rather than what they are capable of, distinguishing it from DNA-based techniques [48]. Its emerging relevance in dairy microbiome research has also been noted [27].

However, Gouveia et al. (2020) [34] identify persistent challenges such as low microbial biomass, interference from host proteins, and the lack of curated goat milk-specific protein databases. There are improved analytical pipelines to enhance protein recovery and interpretation, contributing to more accurate microbial profiling in dairy systems [44,49]. The use of metaproteomics in linking environmental or dietary changes to microbial functionality, reinforcing its relevance for assessing goat milk under different processing or production conditions [29].

Evidence from dairy systems demonstrates the application of metaproteomics to identify proteomic markers of spoilage and fermentation. For example, Soggiu et al., 2016, [50] used metaproteomics to study the impact of lysozyme and clostridial spores on cheese microbiota, identifying microbial proteins associated with spoilage suppression. Similarly, microbial metabolic functions in food waste could be altered by additives such as calcium peroxide, suggesting that similar functional shifts may occur in goat milk during processing or storage [33]. The use of metaproteomics is limited, but a study demonstrated the use of proteomics in goat milk to detect bioactive peptides and microbial enzymes [51], allowing insights into the metabolic roles of microbial communities during fermentation.

Although goat-specific studies are limited, foundational insights into the protein composition of goat milk have been provided [52]. Their work highlights the challenge of high host-protein content, which can interfere with the detection of microbial signals, which is an important consideration in metaproteomics workflows. Additionally, Tilocca et al., 2022, [53] underscores the functional contributions of microbial communities in goat cheese from a One Health perspective, emphasising the value of function-based profiling in dairy research. In metaproteomic studies, commonly used bioinformatic tools include MaxQuant [49], MetaProteomeAnalyzer (MPA) [48], and Proteome Discoverer [34]. These tools facilitate protein identification, quantification, and taxonomic/functional assignment.

#### 2.2.5. Metabolomics

Metabolomics enables the quantification and characterisation of small-molecule metabolites that reflect microbial activity and environmental conditions. In dairy systems, this technique links metabolic outputs to fermentation, spoilage, and probiotic functionality [27,54]. The NMR spectroscopy offers excellent structural clarity and reproducibility [55], while LC-MS and GC-MS are widely used due to their higher sensitivity and broader metabolite coverage [54]. Platforms like MetaboAnalyst and KEGG facilitate pathway interpretation and data visualisation [56]. Metabolomics reveal compositional shifts in goat milk under heat stress [14], and comparative metabolomic analyses of goat and cow milk products, emphasising the distinct functional and nutritional attributes of goat milk [57,58]. However, existing gaps remain, including inconsistent sample preparation protocols and the limited availability of goat-specific metabolite libraries [27,59].

Multiple studies have applied metabolomics to goat milk and yoghurt products, consistently identifying short-chain fatty acids, organic acids, and bioactive peptides. For instance, [14] found that heat stress altered the milk metabolome, while [60] linked metabolite changes to starter culture selection and fermentation characteristics. Despite these promising applications, metabolomics in goat milk is still limited by the lack of unified metabolite databases and standardised protocols. Many detected metabolites remain uncharacterised. Future research should focus on correlating specific microbial species with their metabolite outputs to support functional interpretations.

A metabolomic comparison between goat and sheep milk using GC-MS revealed that goat milk is characterised by higher levels of mannose-6-phosphate, isomaltulose, valine, pyroglutamic acid, leucine, and fucose [61]. These compounds are linked to milk composition traits, such as fat and protein content. Similarly, a storage study of goat milk yoghurt detected 129 metabolites using GC-MS, with notable changes observed in pathways like aminoacyl-tRNA biosynthesis and fatty acid biosynthesis over 28 days [35].

In 2024, Gas Chromatography Flame Ionization Detection (GC-FID), Ultra-High Performance Liquid Chromatography–Quadrupole Time-of-Flight Mass Spectrometry (UHPLC-MS-QToF), Gas Chromatography–Triple Quadrupole Mass Spectrometry (GC-QqQ-MS), and Gas Chromatography–Time-of-Flight Mass Spectrometry (GC-ToF-MSt) were used to analyse goat milk kefir under different heat and fermentation treatments. They identified metabolites such as shikimic acid, GABA, and tyramine, which are associated with immune support and cardiovascular health [62].

Additionally, a comprehensive comparison of goat, cow, sheep, and buffalo milk using targeted metabolomics identified distinct metabolite profiles in goat milk, particularly in amino acids, nucleotides, and organic acids, with enrichment in purine and nucleotide metabolism pathways [63].

Metabolomic data analysis commonly utilises KEGG [59], HMDB [55], and MetaboAnalyst [56]. While these tools provide pathway visualisation and statistical outputs, metabolite identification in goat milk remains challenging due to the limited availability of reference libraries specific to goat dairy matrices.

Advancements in microbial profiling have significantly enhanced our ability to study the complex communities in goat milk. Techniques such as amplicon sequencing, shotgun metagenomics, metatranscriptomics, and metabolomics have improved our understanding of the structure and function of microorganisms. These methods help detect spoilage organisms and identify beneficial species. While each method has its strengths and weaknesses, their combined use provides a robust foundation for microbiome research. Together, they offer a comprehensive understanding of microbial diversity and its impact on the safety and quality of milk.

### 2.3. Spoilage and Shelf-Life of Goat Milk

Like any other animal-derived food, goat milk is vulnerable to microbial contamination during production, which can compromise its quality and safety [64]. “Milk spoilage” refers to a range of undesirable quality changes that can be detected by both producers and consumers through indicators such as abnormal flavours, colour shifts, and inconsistent textures [65]. The extent of spoilage is influenced by numerous factors, including storage conditions, handling practices, hygiene, and the intrinsic properties of goat milk, such as its pH, fat content, and protein composition [66].

Microbial contamination significantly contributes to spoilage, affecting the flavour, texture, and nutritional value of goat milk [67,68]. Spoilage organisms such as *Pseudomonas* spp., *E. coli*, and *Staphylococcus aureus* are major contributors to this deterioration [67]. For instance, *Pseudomonas* spp. exhibit proteolytic activity that breaks down milk proteins, leading to bitter flavours and texture [64]. Ma et al., 2018 [69] also identified *Bacillus* and *Cronobacter* spp. in goat milk. Furthermore, their study highlighted inhibitory microbial interactions, such as a negative correlation between *Lactococcus lactis* and *Bacillus cereus*, which may influence spoilage dynamics.

Spoilage of milk by microorganisms occurs due to the enzymatic degradation of fats, proteins, and carbohydrates by the microbes or their secreted enzymes [67]. In cheese production, psychrotrophic bacteria cause tainting and reduced yield by breaking down casein, resulting in nutrient loss into whey rather than curd formation [70]. As with cow and sheep milk, goat milk is primarily spoiled by *Pseudomonas* and *Bacillus*, which produce thermostable enzymes that degrade milk components, leading to off-flavours, bitterness, and texture issues [65,70].

Spoilage of raw goat milk is predominantly driven by psychrotrophic bacteria, particularly species of *Pseudomonas* and *Acinetobacter*, which proliferate under refrigeration conditions. These microorganisms secrete extracellular enzymes, primarily proteases and lipases, that hydrolyse casein and milk lipids, leading to undesirable sensory changes such as bitterness, rancidity, sedimentation, and a reduction in shelf life [64]. Among these, *Pseudomonas fluorescens* is recognised as a dominant psychrotroph in raw goat milk, notable for producing heat-resistant proteolytic and lipolytic enzymes that remain active post-pasteurisation, thereby compromising the quality of dairy products [71]. Similarly, *Acinetobacter* spp. contributes to spoilage through the production of thermostable enzymes capable of degrading milk constituents, adversely affecting both the sensory and structural integrity of dairy products [72]. Their metabolic adaptability and environmental resilience further exacerbate spoilage by altering the biochemical composition of milk, thereby diminishing its safety and consumer acceptability [73]. These findings underscore the critical importance of stringent cold-chain management and early microbial profiling to mitigate spoilage risks in raw goat milk.

Yeasts and moulds may also contaminate dairy products, causing visible spoilage, off-flavours, and structural defects. Notable fungal species include *Candida* spp., *Geotrichum candidum*, and *Penicillium* spp., which contribute to surface spoilage, pigmentation, and the production of mycotoxins in extreme cases, thereby posing potential safety concerns. For instance, *Geotrichum candidum* has been associated with a decrease in diacetyl content in cottage cheese, leading to flavour defects [74]. Additionally, *Penicillium* species such as *P. commune* and *P. bialowiezense* are commonly found in spoiled dairy products and can produce off-flavours and mycotoxins [75,76].

Lactic acid bacteria (LAB), such as *Lactobacillus* and *Streptococcus*, naturally occur in milk and may contribute to both desirable fermentation and spoilage. While beneficial in controlled fermentation, excess acid production by LAB can lead to curdling and sourness. Enterobacteriaceae like *Escherichia* and *Klebsiella* are indicators of poor hygiene and produce gases and odours. Spore-forming bacteria such as *Clostridium* and *Bacillus* species remain particularly problematic, as they can survive pasteurisation and cause spoilage defects such as late blowing in cheese.

Like any other animal-derived food, goat milk is vulnerable to microbial contamination during production, which can compromise its quality and safety [64]. Spoilage is primarily driven by microbial activity, enzymatic degradation, and physicochemical changes, all of which affect the product’s shelf life. Factors such as hygiene during milking, processing, and storage conditions significantly influence spoilage rates and determine the overall quality of the milk [69]. Table 4 includes the main spoilage bacteria, their impacts on milk quality, and relevant research supporting these effects.

Temperature plays a crucial role in microbial proliferation and enzymatic activity. Studies have shown that proteolytic activity increases with higher temperatures, peaking at 25 °C [64]. Similarly, elevated storage temperatures accelerate microbial growth, leading to faster spoilage [67]. Proper refrigeration and sanitary handling are essential to reducing contamination levels and extending shelf life.

Modern preservation methods aim to extend goat milk’s shelf life without compromising its quality. High-pressure processing (HPP), for instance, has shown success in inactivating spoilage organisms while preserving the sensory and nutritional qualities of milk. HPP treatment at 600 MPa for 7 min extended the shelf life of goat milk up to 22 days at 8 °C, outperforming pasteurisation, which often leads to pH decline and increased microbial counts over time [68]. Other emerging techniques include microfiltration, active packaging, and the application of bacteriocins, which are antimicrobial peptides produced by LAB, which have shown promise in targeting spoilage organisms while avoiding chemical preservatives.

Advanced -omics techniques such as metagenomics, metabolomics, metaproteomics, and metatranscriptomics are increasingly being used to study spoilage processes. These tools allow for comprehensive characterisation of microbial communities, their metabolic activities, and gene expression profiles during spoilage. Metagenomics can identify unculturable spoilage microbes, while metabolomics helps in detecting spoilage-related by-products such as volatile organic compounds. Together, these approaches offer deeper insights into the spoilage ecosystem, facilitating the development of targeted interventions and preservation strategies.

Despite advancements in detection and preservation, significant gaps remain in spoilage research. Studies tend to focus on bacterial spoilage, while fungal spoilage in goat milk remains underexplored. Additionally, real-time profiling and monitoring of spoilage dynamics under informal or small-scale production settings are limited. Improved microbial profiling methods, such as combining next-generation sequencing with digital PCR, could enable earlier detection of spoilage organisms and better prediction of shelf life. There is also a need for integrative, system-level approaches that combine microbial ecology, product formulation, and processing technologies to minimise spoilage and sustainably improve milk quality.

Unlike pasteurisation, which can lead to increased microbial counts and acidity changes over time, HPP-treated milk maintains a stable pH throughout storage [68]. While pasteurisation remains a widely used method, it does not eliminate spoilage risks, emphasising the need for strict hygiene practices throughout production and distribution [67].

Proper preservation techniques and mitigation strategies are crucial for extending the shelf life of goat milk and minimising spoilage. Storage control and improved sanitation are key measures to limit bacterial contamination. Ma et al., 2018 [69] highlighted the need for enhanced hygiene practices throughout the production and processing stages. Their study also suggested that combining advanced detection methods, such as PacBio SMRT sequencing and droplet digital PCR (ddPCR), could improve the accuracy of spoilage organism identification in goat milk powder. The promise of third-generation sequencing and high-precision PCR techniques has been brought to light by recent developments in microbial identification. Full-length 16S rRNA profiling is made possible by PacBio SMRT sequencing, which provides strain-level resolution of spoilage microbes. Similarly, low-abundance spoilage bacteria like Bacillus cereus and Cronobacter spp. may be detected quantitatively and with great sensitivity using droplet digital PCR (ddPCR). These technologies are especially helpful for monitoring microbial loads in processing facilities in real time and for powdered milk surveillance [69].

Effective monitoring and control of spoilage-associated bacteria, particularly *Pseudomonas* spp., are essential for maintaining milk quality [64]. High-pressure processing (HPP) has emerged as a promising alternative to traditional pasteurisation, demonstrating greater efficiency in microbial reduction while preserving the nutritional and sensory qualities of milk [68]. However, maintaining strict hygiene standards during processing is necessary to prevent contamination by *Bacillus cereus*, which poses spoilage risks in both HPP-treated and pasteurised milk. While pasteurisation and HPP remain widely used methods, there is a growing need for novel preservation strategies, including next-generation sequencing (NGS)-based approaches, to enhance microbial safety without compromising milk quality [67].

### 2.4. Goat Milk Microbiota Probiotic Potential

The microbial communities in goat milk, particularly lactic acid bacteria (LAB), offer significant potential for developing functional fermented dairy products. Several probiotic strains have been identified in goat milk that not only support fermentation but also contribute to human health. For example, *Lactobacillus pentosus*, *Lactobacillus plantarum*, *Streptococcus thermophilus*, *Streptococcus bovis*, *Lactococcus lactis*, and *Enterococcus faecium* are recognised as industrially beneficial strains [77]. These microbes exhibit acid tolerance and lactic acid production, which are crucial for survival in the gastrointestinal tract and successful application in dairy fermentation.

In South African Saanen goat milk, *Lactobacillus plantarum* and *Pediococcus acidilactici* have been identified as predominant probiotic strains [8]. These strains not only possess strong acid and bile salt tolerance but also show adhesion to intestinal epithelial cells and antimicrobial activity against pathogens such as *Escherichia coli* and *Staphylococcus aureus*. These features highlight their importance in promoting gut health and maintaining microbial balance. However, Further research is necessary to determine if the microbial communities associated with specific South African goat breeds exhibit probiotic traits, including acid tolerance, adhesion capability, and antimicrobial activity.

Beyond gastrointestinal benefits, probiotics derived from goat milk may help manage metabolic disorders such as obesity and metabolic syndrome with chronic low-grade inflammation. *Lactobacillus* strains have been associated with cholesterol-lowering properties and inhibition of enzymes such as α-glucosidase, suggesting their potential in addressing obesity and hyperglycaemia [78]. These findings open avenues for using goat milk probiotics in functional foods aimed at disease prevention and health promotion.

Additionally, probiotics found in fermented goat milk, such as *Lactobacillus acidophilus* and *Bifidobacterium animalis*, have been shown to positively influence gut microbiota composition and metabolic activity [79]. A goat milk-based diet has been demonstrated to significantly improve fasting glucose levels, glucose tolerance, and pancreatic histological architecture in murine models of type 2 diabetes, indicating a potential role in glycaemic regulation [80]. These metabolic benefits are partially attributed to modulations in gut microbial activity, notably through enhanced production of short-chain fatty acids (SCFAs). Probiotic constituents inherent to goat milk have been shown to elevate SCFA concentrations, thereby supporting gut metabolic function and optimising microbial fermentation processes [81]. These microbial alterations are linked to improved intestinal barrier function and reduced gut permeability, both of which are critical in mitigating systemic inflammation and preventing translocation of pathogenic microorganisms [82].

Probiotics derived from fermented goat milk exhibit immunomodulatory properties through interactions with host immune cells and signalling pathways [79]. Notably, the consumption of fermented goat milk has been implicated in the recovery of iron-deficiency anaemia, an effect mediated by modulation of the gut microbiota and enhancement of intestinal barrier integrity, both of which are essential for maintaining immune homeostasis [83]. In addition, extracellular vesicles isolated from goat milk have demonstrated the capacity to modulate Toll-like receptor (TLR) signalling and influence cytokine secretion in intestinal epithelial cells, suggesting a direct mechanism of communication between microbial-derived components and host immune responses [83]. These findings highlight the systemic impact of bioactive compounds in goat milk on innate immune responses [82].

From a processing perspective, these probiotic strains significantly contribute to the fermentation process. They enhance the nutritional and sensory qualities of fermented products and increase product safety by inhibiting spoilage and pathogenic microorganisms, including *Salmonella* and *Listeria* species [8,77]. Their lactic acid production helps regulate product pH, improving shelf life and consumer safety.

However, the application of goat milk probiotics faces limitations. One challenge is the variability in goat milk composition across breeds, lactation stages, and environmental factors, which may affect microbial activity and product consistency [79]. Additionally, sensory factors, especially the characteristic “goaty” flavour, can limit consumer acceptance of fermented goat milk products. Strategies such as selecting specific strains that mitigate unfavourable flavours or combining goat milk with other ingredients to improve taste profiles may offer practical solutions.

Depending on the product composition, processing technique, and geographical preferences, sensory investigations have revealed a range of reactions. Goat milk, for instance, has been demonstrated to enhance palatability and lessen off-notes when combined with fruit extracts or cow milk [79]. Furthermore, certain strains of *Lactobacillus* can modify volatile profiles during fermentation, increasing fragrance and decreasing bitterness [47]. These results highlight the necessity of combining sensory optimisation techniques with microbial augmentation to increase consumer acceptance of useful goods made from goat milk.

A key gap in current research is the limited number of in vivo studies validating the functional and health-promoting benefits of goat milk probiotics. While in vitro studies show promise, large-scale clinical trials are needed to confirm these effects in human populations. Additionally, there is scope to explore more diverse probiotic strains, particularly those with bioactive properties beyond gut health, such as immune modulation or anti-inflammatory effects.

Overall, goat milk harbours a diverse array of probiotic strains that enhance fermentation and safety, and hold promise for functional food development. Addressing sensory challenges and validating health claims through clinical research will be critical for harnessing their full potential. Future studies should also investigate the synergistic effects of probiotics when combined with other bioactive components in goat milk, contributing to the advancement of innovative dairy products that support both health and consumer demand.

## 3. Methods

This systematic review was conducted following PRISMA (Preferred Reporting Items for Systematic Reviews and Meta-Analyses) guidelines. A comprehensive literature search was performed across ScienceDirect, PubMed, and Google Scholar databases using combinations of keywords such as “metagenomics”, “metabolomics”, “metaproteomics”, “metatranscriptomics”, “next-generation sequencing”, “caprine milk”, “goat milk microbiota”, “probiotic”, and “spoilage.” The inclusion criteria encompassed English-language studies published between 2000 and 2025 that employed high-throughput sequencing or next-generation approaches to investigate microbial communities in goat milk or its products. Studies relying solely on culture-based methods or examining non-dairy microbiomes were excluded.

**Figure 5:**
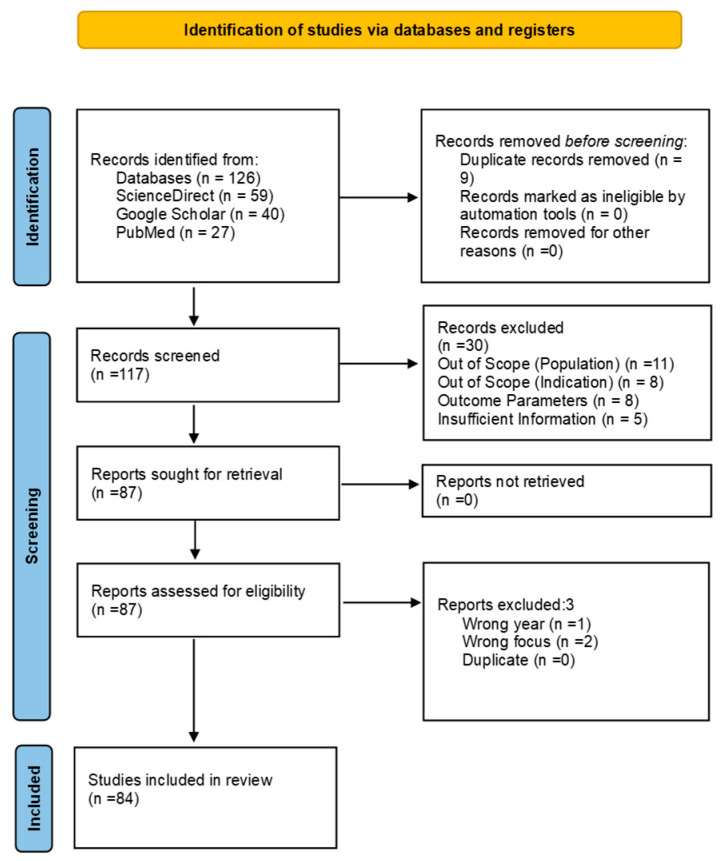
PRISMA flow diagram adapted from Page et al. (2021) [84].

A total of 126 records were identified through database searches. After removing 9 duplicates, 106 records remained for title and abstract screening. Following this, 30 records were excluded. The full texts of 87 reports were then retrieved and assessed for eligibility. Of these, 3 were excluded for not meeting the inclusion criteria. Ultimately, 84 studies were included in this review. The selection process is illustrated in the PRISMA flow diagram (Figure 5). Data extraction focused on the microbial profiling techniques used, key findings regarding microbial diversity and functionality, and methodological strengths and limitations. The quality of the included studies was assessed based on sample size, experimental design, and reproducibility of analytical approaches.

Additionally, only studies that met the specified inclusion criteria and discussed strategies to extend the shelf life of goat milk using high-throughput sequencing techniques were considered.

These investigations included recent techniques like 16S rRNA, metatranscriptomics, metabolomics, shotgun metagenomics, and metaproteomics. This review employs a systematic and comparative analytical approach, evaluating each microbial profiling technique based on its applications, advantages, limitations, and contributions to goat milk’s microbial diversity and functional properties. Tables summarising microbial profiling techniques and key microbial genera were revised to emphasise their relevance to goat milk, highlighting key insights into microbial diversity, functional properties, and probiotic potential.

Every study that was included in this review was evaluated for methodological quality, including sample size, sequencing technique, metadata reporting, and repeatability, in accordance with PRISMA guidelines. Some studies lacked clarification regarding sequencing depth or quality control techniques [6], while others with fewer than 10 samples [3] were identified as having inadequate statistical power. We now draw attention to these restrictions where they are pertinent to the conversation. For instance, due to the lack of breed-specific data and potential environmental contamination, the results of Enterobacter dominance in some raw milk samples need to be taken with caution. Understanding these methodological variations guarantees that this synthesis captures the robustness and consistency of the supporting data in addition to microbiological trends.

## 4. Conclusions and Recommendations

This systematic review aimed to evaluate emerging microbial profiling techniques applied to goat milk, particularly in identifying spoilage organisms, probiotic strains, and microbial diversity. Analysing 73 studies, it is evident that techniques such as amplicon sequencing, shotgun metagenomics, metatranscriptomics, metabolomics, and metaproteomics provide valuable insights into the functional roles of microbial communities. These methods have enhanced our understanding of how specific taxa like *Lactobacillus*, *Pseudomonas*, and *Staphylococcus* impact goat milk safety, quality, and fermentation potential. The review confirms that these techniques are crucial for advancing dairy microbiology and meeting the growing demand for probiotic-rich and minimally processed dairy products.

Although several reviews have explored microbial profiling in dairy products, most concentrate on bovine milk or general ruminant matrices, with limited focus on dairy goats. The existing literature often centres on single techniques like 16S rRNA gene sequencing, overlooking the expanding array of advanced tools such as shotgun metagenomics, metaproteomics, metabolomics, and metatranscriptomics. This review offers a unique contribution by comparatively synthesising these emerging methods specifically in the context of goat milk, addressing both spoilage-related and probiotic microbial communities. Moreover, systematic reviews employing rigorous methodologies to assess microbial diversity in goat milk are rare, especially those integrating multi-omics approaches. By spotlighting dairy goats and incorporating diverse high-throughput technologies, this review fills a critical gap and provides a more holistic understanding of microbial dynamics in this underrepresented species.

Techniques such as 16S rRNA gene sequencing, shotgun metagenomics, and culturomics enable high-resolution identification of both beneficial and harmful microbes. Their application allows for early detection of spoilage organisms and pathogens, supporting improved hygiene practices, extended shelf life, and safer consumption. Functional insights gained through metabolomics and metatranscriptomics further elucidate microbial activity, including the production of bioactive compounds or spoilage metabolites. Integrating these tools enhances our understanding of microbial dynamics throughout processing and storage, aiding stakeholders in implementing targeted interventions. Together, these techniques shift microbial profiling from basic enumeration to a more holistic and predictive approach, informing quality control, product development, and regulatory compliance in the dairy sector.

The composition and function of the milk microbiome are influenced by multiple interacting factors, including host physiology, environmental conditions, and microbial interactions. These interconnected elements collectively determine milk quality and safety. For instance, microbial dysbiosis, particularly during mastitis, has been linked to reduced milk quality and increased risk of contamination. Pathogenic species such as *Staphylococcus aureus* and *Escherichia coli* tend to dominate in dysbiotic states, whereas beneficial genera like *Lactobacillus* and *Bifidobacterium* are depleted [27]. These shifts not only affect milk composition but also its suitability for consumption and processing. The integration of multi-omics approaches allows for a more holistic understanding of these dynamics, enabling better diagnostics, quality control, and intervention strategies in dairy systems.

Despite these advancements, notable limitations remain in both methodological practices and the existing body of literature. Many studies lack standardisation in experimental design, sequencing protocols, and sample handling, making cross-study comparisons challenging. Additionally, goat-specific reference databases for DNA, RNA, proteins, and metabolites are limited, reducing taxonomic and functional resolution. Omics techniques, though powerful, still face challenges such as RNA instability, low microbial biomass, and host interference, which can bias results. Furthermore, there is a significant gap in translating these findings into large-scale, longitudinal studies that account for breed variation, lactation stages, and environmental influences.

Future research should focus on standardising microbial profiling workflows and expanding goat-specific bioinformatic resources. There is a clear opportunity to explore microbial interactions over time and across different production systems to better predict spoilage, optimise fermentation, and develop functional dairy products. For the industry, these insights can support the selection of robust starter cultures, improve preservation strategies, and reduce product waste. Additionally, regulatory bodies can use data from microbial profiling to refine microbial quality standards, guide the development of goat milk-specific food safety protocols, and enhance real-time monitoring practices. Together, these advances will contribute to safer, more efficient, and more innovative goat milk production.

## Figures and Tables

**Table 1:** A comparison of microbial profiling techniques in goat milk.

Technique	Principle	Advantages	Limitations	Reference
Metagenomics	Sequencing of microbial DNA from environmental samples	Identifies unculturable species, provides comprehensive microbial diversity	Requires advanced bioinformatics	[12]
Metabolomics	Analysis of metabolites in milk	Identifies biomarkers for quality and spoilage with high sensitivity	Complex data interpretation requires specialised equipment	[13]
Metatranscriptomics	Sequencing of RNA to study gene expression in microbial communities	Provides insights into active microbial functions and metabolic pathways	High-cost, complex data analysis	[16]
Next-Generation Sequencing (NGS)	High-throughput sequencing of microbial genomes	Hugh’s resolution identifies strain-level diversity	Expensive, requires computational resources	[17]

**Table 2:** Summary of key microbial genera found in goat milk, their functions and relevant references.

Bacterial Group	Genera/Species	Function	Potential Impact	Reference
Beneficial bacteria	*Lactococcus*, *Lactobacillus*, *Leuconostoc*	Plays a crucial role in fermentation, producing lactic acid and enhancing probiotic potential.	Enhances fermentation, improves probiotic potential, and extends shelf-life	[5,6]
	*Bifidobacterium*, *Curtobacterium*	Improves the content of short-chain fatty acids and medium-chain fatty acids in fermented milk.	Contributes to gut health, potential probiotic	[5,6]
Spoilage bacteria	*Pseudomonas*, *Bacillus*, *Staphylococcus*	Produces extracellular enzymes that digest milk proteins and fats, leading to spoilage.	Causes spoilage, impacts milk safety and quality	[3,7]
	*Enterobacter*, *Pseudomonas*	Commonly found in raw milk; its role in spoilage and fermentation is under investigation.	Can spoil milk, impacts quality	[3,19]
Pathogenic bacteria	*Escherichia.* sp, *Staphylococcus aureus*	Associated with milk safety concerns, but also part of the natural microbiota.	Causes health risks, spoilage issues	[7,20]
	*Mycoplasma* sp.	Pathogenic, spoilage	Associated with mastitis, reduces microbial diversity	[20]

**Table 4:** Key spoilage bacteria, their effects on goat milk quality.

Spoilage Bacteria	Effects on Milk Quality	Reference
*Bacillus cereus*	Potential food spoilage and foodborne illness risk, quantified at 6.3 × 10^4^ copies/g in goat milk powder.	[69]
*Cronobacter* spp.	Food safety risk, quantified at 1.0 × 10^4^ copies/g in goat milk powder.	[69]
*Pseudomonas fluorescens*	Proteolytic activity leading to spoilage; observed at refrigeration (7 °C) and ambient temperatures (25–35 °C).	[64]
*Pyschrotrophic bacteria*	Spoilage in pasteurised goat milk, exceeding permissible limits during storage.	[68]
*Pseudomonas* spp.	Causes flavour, texture, and nutritional degradation in milk and milk products.	[67]
*E. coli*	Contributes to spoilage, altering flavour and safety.	[67]
*Staphylococcus aureus*	Spoilage and potential foodborne illness risk.	[67]
